# Modelling the Protective Efficacy of Alternative Delivery Schedules for Intermittent Preventive Treatment of Malaria in Infants and Children

**DOI:** 10.1371/journal.pone.0018947

**Published:** 2011-04-20

**Authors:** Matthew Cairns, Azra Ghani, Lucy Okell, Roly Gosling, Ilona Carneiro, Francis Anto, Victor Asoala, Seth Owusu-Agyei, Brian Greenwood, Daniel Chandramohan, Paul Milligan

**Affiliations:** 1 London School of Hygiene and Tropical Medicine, London, United Kingdom; 2 Imperial College, London, United Kingdom; 3 Navrongo Health Research Centre, Navrongo, Ghana; 4 Kintampo Health Research Centre, Kintampo, Ghana; University of Oxford, Viet Nam

## Abstract

**Background:**

Intermittent preventive treatment in infants (IPTi) with sulfadoxine-pyrimethamine (SP) is recommended by WHO where malaria incidence in infancy is high and SP resistance is low. The current delivery strategy is via routine Expanded Program on Immunisation contacts during infancy (EPI-IPTi). However, improvements to this approach may be possible where malaria transmission is seasonal, or where the malaria burden lies mainly outside infancy.

**Methods and Findings:**

A mathematical model was developed to estimate the protective efficacy (PE) of IPT against clinical malaria in children aged 2-24 months, using entomological and epidemiological data from an EPI-IPTi trial in Navrongo, Ghana to parameterise the model. The protection achieved by seasonally-targeted IPT in infants (sIPTi), seasonal IPT in children (sIPTc), and by case-management with long-acting artemisinin combination therapies (LA-ACTs) was predicted for Navrongo and for sites with different transmission intensity and seasonality. In Navrongo, the predicted PE of sIPTi was 26% by 24 months of age, compared to 16% with EPI-IPTi. sIPTc given to all children under 2 years would provide PE of 52% by 24 months of age. Seasonally-targeted IPT retained its advantages in a range of transmission patterns. Under certain circumstances, LA-ACTs for case-management may provide similar protection to EPI-IPTi. However, EPI-IPTi or sIPT combined with LA-ACTs would be substantially more protective than either strategy used alone.

**Conclusion:**

Delivery of IPT to infants via the EPI is sub-optimal because individuals are not protected by IPT at the time of highest malaria risk, and because older children are not protected. Alternative delivery strategies to the EPI are needed where transmission varies seasonally or the malaria burden extends beyond infancy. Long-acting ACTs may also make important reductions in malaria incidence. However, delivery systems must be developed to ensure that both forms of chemoprevention reach the individuals who are most exposed to malaria.

## Introduction

Intermittent preventive treatment of malaria in infants (IPTi) consists of a full therapeutic course of antimalarial drugs given at the time of Expanded Program on Immunisation (EPI) vaccination in infancy. EPI-linked IPTi using sulphadoxine-pyrimethamine (SP) provides a protective efficacy (PE) of approximately 30% against clinical episodes of malaria during infancy [1]. EPI-IPTi has been extensively evaluated and has been shown to be safe, well-tolerated, acceptable and highly cost-effective [1], [2], [3], [4]; EPI-IPTi is now recommended as policy by WHO in areas with a high burden of malaria in infancy and a low prevalence of SP resistance [5]. However, some uncertainty remains about whether the currently recommended EPI-linked IPTi strategy could be adapted to certain settings to improve efficacy, particularly where malaria transmission is seasonal or where a large part of the malaria burden occurs outside infancy. Therefore, it would be helpful to know how alternative forms of chemoprevention would compare to IPTi delivered through EPI.

One potential alternative to EPI-IPTi is seasonally-targeted intermittent preventive treatment, where IPT is delivered at the time of peak malaria transmission rather than at the time of age-based EPI vaccination contacts. Seasonal IPT could be restricted to infants alone, or extended to include children above one year of age. A number of studies have shown that seasonal IPT in young children is very effective in preventing malaria when given at the time of peak transmission [6], [7], [8], [9]. If the challenges of delivering the intervention outside the structure of the EPI can be met, there are potentially two major advantages of seasonally-targeted IPT. Firstly, because the major benefit of IPT is probably short-term chemoprophylaxis [10], [11], [12], delivering courses of IPT at the time of greatest malaria risk will avert more cases of malaria. Secondly, a delivery system set up to deliver IPT outside the EPI would be able to include young children in addition to infants, and thus help to tackle the malaria burden outside infancy. Young children may suffer more severe malaria than infants in certain epidemiological settings [13], [14], and the malaria burden in infants may decrease further relative to that in children in the context of falling transmission intensity and other control efforts [15], [16], [17].

Another possible means to prevent malaria through the use of antimalarials would be to use long-acting drugs for the treatment of malaria cases. Both malaria infection and clinical malaria are overdispersed, meaning that some children suffer multiple malaria episodes while others around them suffer no malaria. A clinical malaria episode is thus a strong predictor of subsequent episodes [18], [19]. New long-acting artemisinin combination therapies (LA-ACTs), which include a long-acting partner drug (e.g. piperaquine, pyronaridine or mefloquine) may offer post-treatment prophylaxis to the children who most need protection from malaria [20]. Reductions in overall malaria incidence have been seen when ACTs with long-acting partner drugs were introducted in Zanzibar and in South East Asia [21], [22]. Where the malaria burden is seasonal, use of LA-ACTs for malaria case management may also protect children from further episodes of malaria at the time of year when they are most at risk. Where LA-ACTs are widely available for case management, this could therefore prevent as well as treat malaria.

In this study, a mathematical model was developed to investigate the protective efficacy of IPTi delivered at EPI vaccination contacts in infancy, and three alternatives, namely seasonal IPT in infants (sIPTi), seasonal IPT in children (sIPTc) and the use of long-acting ACTs for case-management of malaria (LA-ACTs). This was undertaken first for a specific site in Ghana for which detailed contemporaneous data on malaria incidence in children <24 months of age, entomological inoculation rate (EIR), and SP-IPTi efficacy were available. The site represents an area where malaria transmission is highly seasonal, although there is some transmission throughout the year. Under such conditions, the advantages of seasonal targeting are less obvious than in areas with a very short transmission season each year, such as many areas of the Sahel [23]. The model was then extended to consider the impact of these different strategies in sites with a range of seasonality and transmission intensity patterns.

## Methods

### Data

This study used data from the Navrongo IPTi study, which took place in Kassena-Nankana District (KND), Ghana between 2000 and 2004. The IPTi trial is described in detail elsewhere [24]. In brief, four courses of SP or placebo were given to infants at 3, 4 and 9 months of age at the time of an EPI contact and at 12 months during a growth-monitoring visit. Passive case detection of malaria continued until children reached two years of age. This analysis therefore focuses on children less than 24 months of age. Incidence rates of malaria by calendar month in the intervention and control groups were calculated from the IPTi trial data. Entomological inoculation rate (EIR) in each calendar month was provided by an entomological study which took place in KND during the period of the trial (Anto et al. [25], unpublished).

### Model development

A set of deterministic compartmental models was developed. These are specified as sets of differential equations which describe changes over calendar time in the number of individuals in each compartment. The model equations are described in detail in Supporting Information S1. The infection status of the human population (<24 months of age) in calendar time is described by a compartmental model. For simplicity, there is no analogous mosquito component: this is replaced with a function representing the entomological inoculation rate, with monthly values estimated from the recorded entomological data.

**Figure 1:**
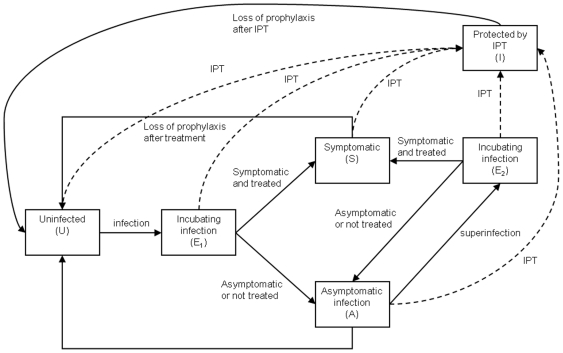
Overview of IPT model structure. The main states and transitions in the model are shown. IPT is indicated by dashed lines. There are multiple analogous components in the model: the diagram represents one combination of age, intervention and exposure group.

In the model, children can be in one of the following states: uninfected (U), previously uninfected incubating infection (E_1_), previously asymptomatic/untreated incubating superinfection (E_2_), asymptomatic or untreated parasitaemia (A), symptomatic and treated (S) or protected by IPT (I) (figure 1). Children enter the model at 2 months of age, the age of recruitment in the trial, and are assumed to be uninfected at this time. Uninfected children become infected at a rate which depends on the entomological inoculation rate and the probability that an infective bite results in infection. Infected individuals transfer to E_1_, which represents the incubating state. A proportion of newly infected children become symptomatic at the end of the incubation period and move into compartment S. The remainder develop asymptomatic parasitaemia and transfer to A. For simplicity it is assumed that once asymptomatic, children do not subsequently develop symptoms unless they are reinfected. Children with asymptomatic parasitaemia recover via three additional compartments before returning to the susceptible state. The use of four compartments for the infected state makes the distribution of time spent infected more realistic than if a single compartment with a constant rate of recovery was used: the total duration of protection then follows a gamma distribution rather than an exponential distribution [26]; this also allows a simple means of including the effect of superinfection [27]. Children who are superinfected enter an incubating compartment, E_2_, after which a proportion develop symptoms and are treated (moving from E_2_ into S), or remain asymptomatic and return to A_1_. In the simplest case, the probability of developing symptoms was assumed to be the same for both newly infected children and children with superinfections; this assumption was relaxed in later versions of the model. Acquisition of clinical immunity with increasing age is accounted for by allowing the proportion of infected children that develop clinical symptoms to vary between age groups. To incorporate effects of maternally-derived immunity, the probability that an infectious bite results in infection is assumed to be lower early in infancy than later (see Supporting Information S1 for details).

Because the intention was to validate the model with trial data, we assumed that all children who had symptomatic infections and who reported to trial staff were treated. In the model, treated children recover faster than untreated or asymptomatic children and receive a period of post-treatment prophylaxis (PTP) when they cannot be reinfected. This is modelled by delaying the return of treated children to the uninfected (and susceptible) compartment, U, via a series of sub-compartments as described above [26]. Chloroquine (CQ) was the antimalarial most commonly used for first line treatment during the Navrongo trial. Post-treatment prophylaxis against erythrocytic stages may last for as little as two weeks where CQ resistance is high [20], as was the case in Navrongo at the time of the trial [28].

This model structure describes the transmission dynamics in the control group of the Navrongo trial. For the intervention group, an identical set of equations is used but with the addition of compartments representing intermittent preventive treatment. The model was stratified into two separate age groups (infants <12 months of age, and children 12–23 months of age), allowing the impact of IPT given to specific age groups to be explored. The model was further stratified to reflect heterogeneity in malaria exposure, assuming that 20% of individuals receive 80% of all infections [13], [29].

**Figure 2:**
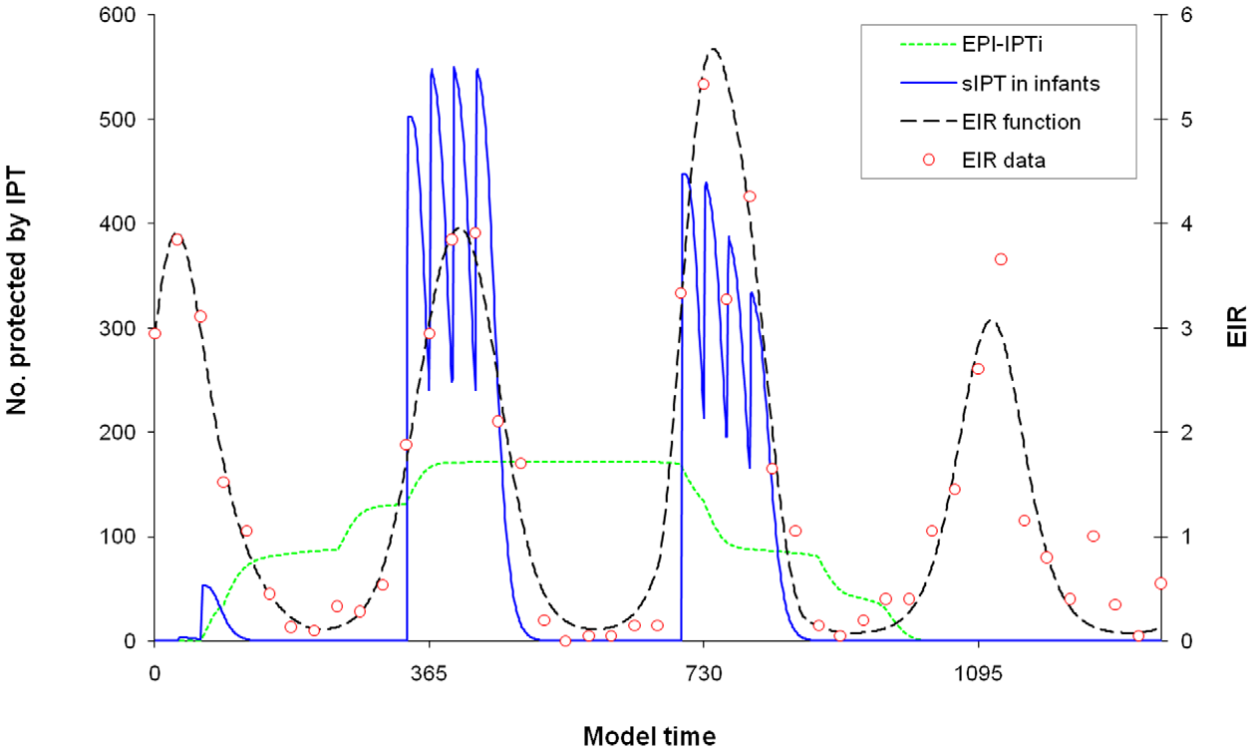
Illustration of EPI-IPT and seasonal IPT in infants. The number of children given IPT with two different strategies: 1) four courses of EPI- IPTi given at 3, 4, 9 & 12 months of age and 2) four courses of monthly seasonal IPT given to all infants regardless of their exact age. The EIR function from the model fitted to EIR data from Navrongo is also shown. Fitting of the EIR function is described in detail in Supporting Information S1.

The proportion of individuals successfully protected by IPT depends on the coverage of IPT and the proportion of IPT courses that are efficacious. It was assumed that a course of IPT would be ineffective in a certain proportion of children who received IPT, either due to drug resistance of *P. falciparum*, receipt of an insufficient dose of SP, vomiting of treatment, or other similar reasons. It is assumed that an efficacious course of IPT will clear parasitaemia. In addition to its curative effect, IPT is considered to provide prophylactic protection against new infections. Individuals protected by IPT move into the IPT component of the model and remain in this state for a variable period before returning to U. As for the symptomatic treatment component, the IPT component is modelled with multiple compartments such that the duration spent protected follows a gamma distribution. Implementation of IPT is modelled in one of two ways, reflecting two different delivery strategies: 1) EPI-linked IPT delivered to infants at specific age-based vaccination contacts (EPI-IPTi) and 2) IPT given outside the EPI programme to all infants, regardless of their exact age, as simultaneous mass treatments during the season of highest malaria transmission (sIPT). With IPT given via the EPI, the cohort of infants receives IPT throughout the year as members reach the appropriate age to attend vaccination clinics (for Navrongo this was 3, 4 and 9 months with an additional growth monitoring visit at 12 months of age). With seasonal-IPT, all infants receive IPT at specific times of year according to the peak in transmission (figure 2).

### Parameter estimation and model validation

The model was developed and parameterised to reflect the situation of the Navrongo trial, and parameter values were estimated from data from the trial and trial area before consideration of other settings. Enrolment rate over calendar time is based on trial data. The magnitude and duration of protection against malaria from IPTi were estimated as previously described [10]. The EIR function represents the fluctuations in the force of infection over time, which were estimated using the data collected in KND during the trial period by Anto et al. ([25], unpublished data). Other parameter estimates were obtained from the literature. Key parameters and their values are given in table 1; a full list is given in Supporting Information S1. Cumulative malaria incidence in each month in the model was fitted simultaneously to monthly incidence data from four groups in the trial (infants and one year old children in both the SP and placebo groups). Fitting was carried out using the optimize algorithm in Berkeley Madonna (version 8.3.11, University of California) by varying *θ*, the proportion of infections that are symptomatic and treated, in order to maximise the binomial log-likelihood function. When children in KND were followed actively after radical cure, around 30–35% developed symptoms upon first infection [30]. The proportion of symptomatic infections detected passively is likely to be somewhat lower, because not all children with malaria will present for treatment. Values of *θ* estimated by fitting the model were 23.7% for infants and 20.4% for one year old children, both of which are biologically plausible. A sensitivity analysis was carried out by varying the parameters of the model within the range indicated in the Supporting Information S1.

**Table 1:** Descriptions and values of key model parameters.

Parameter description (units)	Values from literature/data and source	Best estimate (range)
Entomological inoculation rate	Function fitted to entomological data (for Navrongo) or sinusoidal function (other settings)	-
Probability an infective bite results in infection in a child	0.006 (High EIR), 0.07 (Low EIR) [41]	0.02 (0.005, 0.1)
	0.026–0.073 (EIR = 1 per day) [42]	
	0.03–0.13 [43]	
	0.1 [44]0.2	
Rate individuals are infected	Varies with time: determined by EIR and probability infective bite results in infection	-
Mean duration of incubation period (days)	12 (9–14) [45]	10 (7, 14)
	9 [46]	
Mean duration of asymptomatic infection if untreated (days)	23 (highly endemic) [47]	100 (50, 200)
	73 (<18 months of age)160 (18–23 months) [48]	
	210 (176–256) [49]	
	210 (183–236) [50]	
Proportion of new infections that are symptomatic	0.31 (KND, dry season),0.34 (KND, wet season) [30]0.44 (Kenya) [51]	
% infections symptomatic *and treated* in infants	Parameter fitted by the model	23.7% (5, 35)
% infections symptomatic *and treated* in 1 year olds	Parameter fitted by the model	20.4% (5, 35)
Mean duration of prophylaxis after treatment for malaria (days)	CQ during Navrongo trial14-day ACPR 47% [28]14 days PTP after CQ treatment [20]	14 (7, 21)
IPT Coverage	0.92 Navrongo data	0.92 (0.5, 0.95)
IPT Efficacy	14 day ACPR in KND: 0.78 [28]. Best estimate from model fitting.	0.9 (0.5–0.95)
Mean duration of prophylaxis after IPT (days)	Protection for 28–42 days (SP) [10]Up to 60 days [52]Gamma distribution fitted to data.	30 (20–35)

### Protective efficacy of EPI-IPTi and seasonal IPT in infancy

For all analyses, PE was calculated by comparing the cumulative incidence by 24 months of age predicted by the model in the intervention and control groups (i.e. among those children given EPI-IPTi in addition to normal-case management, and those given only case-management). Firstly, IPTi delivered through the EPI as in the Navrongo trial was compared to a seasonal IPT strategy in which the same number of courses of treatment (four) was delivered to all infants, regardless of their exact age, on a monthly basis during the peak transmission period. Secondly, the PE of different seasonal IPT strategies was investigated by varying the number of IPT courses per year, the timing of IPT courses, and the coverage of each IPT course. Thirdly, the effect of using a longer-acting drug for sIPT or EPI-IPTi on the relative benefit of seasonal targeting was investigated.

### Protective efficacy of seasonal IPT in children

In later versions of the model, seasonal IPT was extended to include children aged 12–23 months in addition to infants. Although previous trials of IPT in children have usually included children under 5 or 10 years of age, because the model is fitted to the Navrongo trial cohort we simulate only the impact of including one additional year of IPT and present protective efficacy at 24 months of age.

### Long-acting ACTs for case-management of malaria

Finally, the effect of a long-acting drug regimen for case management was investigated by varying the duration of protection obtained after treatment for symptomatic malaria. This was done both in the absence of IPT strategies, and alongside EPI-IPTi, sIPTi and sIPTc.

### Extension of the model to other sites

A previous study defined ‘marked seasonality’ as the occurrence of more than 75% of the annual malaria incidence in six months of the year [31]. Using similar logic, seasonality can be considered to vary from 50% of annual incidence occurring within a 6 month period (i.e. stable transmission all year) to 100% (highly seasonal). Simple periodic functions were used to simulate varying degrees of seasonal transmission ranging from 50% to 100% of transmission within 6 months (at 10% intervals). We investigated the protective efficacy of EPI-IPTi, sIPTi and sIPTc under these different seasonality patterns, using two different transmission intensities: high (EIR = 365, i.e. one infectious bite per individual per night, a similar intensity to Navrongo) and low-moderate (EIR  = 10, the lower transmission intensity threshold recommended for implementation of IPTi).

## Results

**Figure 3:**
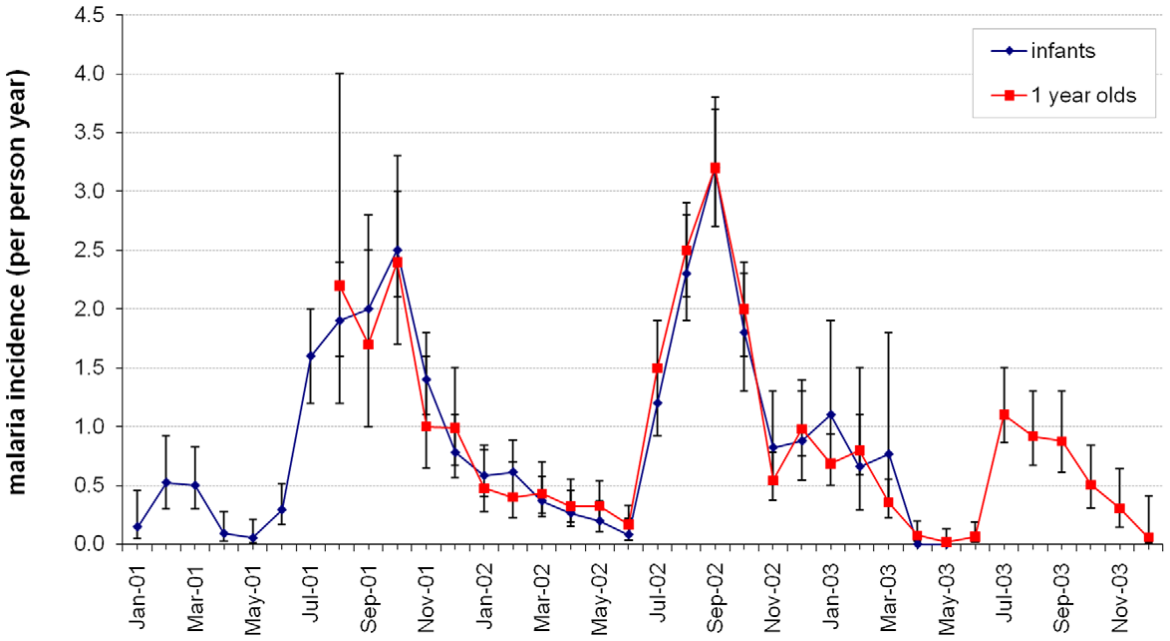
Incidence of clinical malaria in the Navrongo IPTi trial. Incidence of clinical malaria between January 2001 and December 2003 is shown for infants and children 12–23 months of age in the placebo group of the IPTi trial. Error bars indicate 95% confidence interval. Children were enrolled between September 2000– June 2002. Completion of 24 months follow-up ended in June 2004.

Malaria incidence by calendar month in Navrongo is shown in figure 3. Incidence peaks between July and October and tails off gradually into the dry season, corresponding closely with the changes in EIR (figure 2).

### Model validation and sensitivity analysis

**Figure 4:**
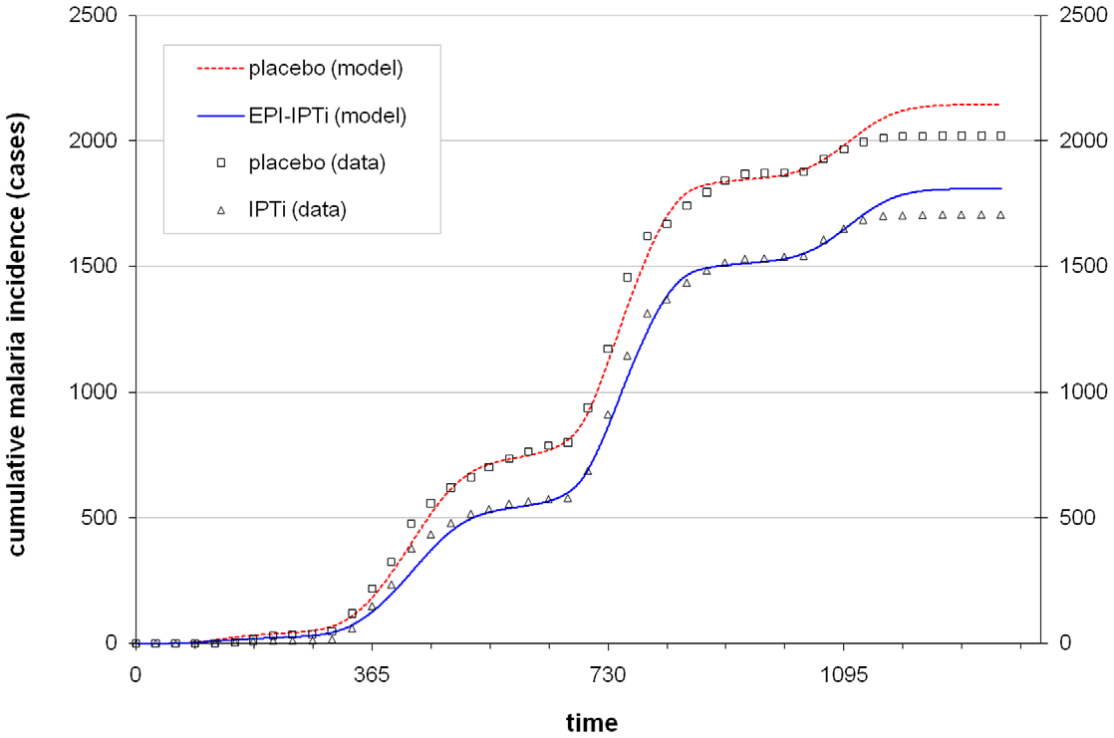
Cumulative malaria incidence predicted by the fitted model. Cumulative malaria incidence over the period of the Navrongo IPTi trial. Points indicate incidence data from the trial; the dotted and solid lines indicate the fitted model predictions the for placebo and EPI-IPTi (IPTi) groups respectively.

Cumulative incidence of clinical malaria observed during the Navrongo trial, and the corresponding values predicted by the model are shown in figure 4. The model predictions agreed well with the observed patterns. In particular, predicted cumulative incidences in infancy were very close to the observed values. The model slightly overestimated cumulative incidence in one year old children, predicting around 100 more cases than actually occurred, but this was similar in both the intervention and control groups. The fitted model predicted protective efficacy for EPI-linked IPT of 25.3% at 15 months of age and 15.6% by 24 months of age, very similar values to those obtained in the clinical trial (24.9% at 15 months and 16.3% at 24 months of age [24]). The sensitivity analysis (Supporting Information S1) indicated that varying the parameter values did affect the point estimates of protective efficacy, but in all cases the relative magnitude of the differences between seasonal IPT and EPI-IPTi remained similar.

### Protective efficacy of EPI-IPT and seasonal IPT in infancy

The model prediction of the efficacy of four courses of seasonal IPT given to infants during the peak transmission season was 26.1% at 24 months of age (table 2). Four courses of monthly seasonal IPT given to infants with coverage of 53% would be equally protective as four courses of EPI-IPTi with coverage of 92% (as in the clinical trial). Assuming the same level of coverage, two seasonally-targeted IPT courses in infancy alone would give similar protection to four courses of IPTi given through the EPI (PE 14.9% and 15.6% respectively). High efficacy would be achievable with multiple courses of sIPT: with six courses of sIPT given monthly to infants, protective efficacy would be 33.1% at 24 months of age.

**Table 2:** Effect of long-acting drug for case management on IPT efficacy.

	PTP from drug used for case-management* (days)	Predicted cases by 24 months		Cases averted by		Total cases averted	Percent averted by IPT	PE of IPT at 24 m (%)	Relative PE of sIPT$ vs EPI-IPTi
		Placebo	SP	Treatment∧	IPT£				
**EPI-IPTi**	14	2145	1810	-	335	335	-	15.6	-
	30	1944	1659	201	286	487	59	14.7	-
	45	1809	1560	336	248	584	42	13.7	-
	60	1705	1488	440	217	657	33	12.8	-
	90	1559	1389	586	169	755	22	10.9	-
**sIPT** **in** **infancy**	14	2145	1584	-	561	561	-	26.1	1.7
	30	1944	1466	201	478	679	70	24.6	1.7
	45	1809	1389	336	420	756	56	23.2	1.7
	60	1705	1331	440	374	814	46	21.9	1.7
	90	1559	1251	586	308	894	34	19.8	1.8
**sIPT in** **children<24 months**	14	2145	1036	-	1109	1109	-	51.7	3.3
	30	1944	987	201	957	1158	83	49.2	3.3
	45	1809	959	336	850	1186	72	47.0	3.4
	60	1705	939	440	766	1206	64	44.9	3.5
	90	1559	912	586	647	1233	52	41.5	3.8

* Mean duration of post-treatment prophylaxis after treatment for a symptomatic malaria episode, 14 days is the value assumed for CQ in Navrongo. For this analysis, duration of post-treatment prophylaxis after IPT was fixed at 30 days.

∧ cases averted relative to a drug for case management with mean duration of PTP of 14 days.

£ Number of cases averted by IPT (difference between cases in placebo group and SP group) with treatment drug of same duration.

$ Relative protective efficacy of seasonal IPT versus EPI-IPTi with same duration of PTP after treatment for symptomatic malaria.

The use of longer-acting drugs for IPT would increase protective efficacy and reduce the advantages of seasonal targeting. However, within the constraints of drugs that are currently available, targeting IPT at the peak in transmission remains necessary to maximise efficacy in seasonal settings. With a hypothetical IPT drug that provided a mean of 90 days prophylaxis, longer than any currently available antimalarial, four courses of EPI-IPTi would provide protective efficacy by 24 months of age of 36.1%; this would be 42.3% with four courses of monthly seasonal IPT in infants.

### Protective efficacy of seasonal IPT in children

The predicted PE of four monthly courses of sIPT given at the peak in transmission during both the first and second years of life was 51.7% between 2–24 months of age.

### Long-acting ACTs for case-management of malaria

Use of a long-acting drug regimen for case management would be expected to reduce the number of malaria cases in both the SP and placebo groups. The PE of IPT would also decrease with increasing post-treatment prophylaxis after curative treatment, because more children would already be protected by their most recent curative treatment at the time they receive IPT (table 2). In model simulations, case management alone using a drug regimen which provides a mean of 45 days of post-treatment prophylaxis (PTP), prevented a similar number of malaria cases to a strategy of EPI-linked IPTi with SP in conjunction with a shorter-acting regimen for case management which provided 14 days PTP. However, IPT of either type (EPI-IPTi or seasonal IPT) in addition to a long-acting drug regimen for case management is more efficacious than either strategy alone. Seasonal IPT in the presence of long-acting regimens for case management retains its relative advantage over EPI-IPTi, though the absolute benefits would become smaller.

### Extension of the model to other sites

Seasonal targeting of IPT given only to infants does not improve efficacy in sites with completely uniform transmission over the whole year, as would be expected (table 3). In sites with a short transmission season of three months, the efficacy of seasonal IPT given each year for two years was predicted to be over 80% at 2 years of age, a finding that agrees with existing trials of seasonal IPT in children (Supporting Information S1). However, the conditions under which seasonal IPT may have advantages over EPI-IPTi are not restricted to scenarios with marked seasonality (table 3). An ability to target children outside infancy would be an important advantage of alternative delivery mechanisms, even in situations where malaria transmission is not seasonal. The advantages observed were consistent in high and low-moderate transmission settings.

**Table 3:** Protective efficacy of EPI-IPTi and seasonal IPT by epidemiological setting.

	Protective efficacy by 24 months of age (%)			Relative protective efficacy	
Transmission in peak 6 months of the year (%)	EPI-IPTi	sIPTi	sIPTc	sIPTi vs.EPI- IPTi	sIPTc vs.EPI-IPTi
**High EIR (365)**					
50	15.8	14.5	28.9	0.9	1.8
60	15.7	16.6	33.0	1.1	2.1
70	15.6	18.9	37.6	1.2	2.4
80	15.4	21.8	43.3	1.4	2.8
90	15.1	25.5	50.7	1.7	3.4
100	14.9	31.8	63.3	2.1	4.3
**Low-moderate EIR (10)**					
50	17.2	15.1	30.8	0.9	1.8
60	17.1	18.3	37.7	1.1	2.2
70	16.9	21.5	44.8	1.3	2.7
80	16.8	24.7	51.9	1.5	3.1
90	16.6	28.0	59.5	1.7	3.6
100	16.5	32.6	71.7	2.0	4.4

Protective efficacy of EPI-linked intermittent preventive treatment in infants (EPI-IPTi) and seasonal IPT under different seasonality and transmission intensity. EPI-IPTi delivered at 3, 4, 9 and 12 months of age. Seasonal IPT consists of four monthly courses of seasonal IPT targeted at the peak in transmission given to infants alone (sIPTi) or to all children <24 months of age (sIPTc).EIR: entomological inoculation rate.

## Discussion

Malaria chemoprevention by intermittent administration of courses of antimalarials such as SP can provide personal protection against sensitive parasites for about one month. The impact of a particular strategy on the malaria burden will depend on the age distribution and seasonal pattern of malaria in a particular situation, the number and timing of treatment courses, the age groups targeted, and the coverage achieved. Our mathematical model helps to explain the likely impact of different chemoprevention strategies in a range of epidemiological settings.

In Navrongo, an area with extended seasonal transmission, four monthly courses of sIPT given only to infants would provide a protective efficacy of 26.1% at 24 months of age. Seasonal IPT would provide similar efficacy to EPI-IPTi even if the coverage achieved by the alternative delivery system was markedly lower. When possible differences in coverage among highly exposed individuals were considered, seasonal IPT was predicted to be more protective in all but the most extreme scenarios (Supporting Information S1). Bojang et al. [32] compared EPI-based and community-based delivery for IPT in children; both achieved equitable coverage with respect to socioeconomic status determined from household assets. Furthermore, our model predicts that seasonally-targeted IPT may be able to provide similar protective efficacy to EPI-IPTi with fewer courses of drugs. Longer-acting drugs for IPT would be expected to improve the efficacy of both EPI-IPTi and seasonal IPT. However, with sensible constraints on the duration of protection, benefits would remain from seasonal IPT rather than EPI-IPTi.

The model simulations predicted that the use of a long-acting regimen for malaria case management would lower overall malaria incidence by providing post-treatment prophylaxis to children who are treated for malaria. Reductions in transmission as a result of ACT use have been predicted by previous modelling studies [27] and have been observed in practice [21], [22]. Our model assumes that only individuals who actually have malaria (and not those who receive treatment for fevers due to other causes) would receive post-treatment prophylaxis, which follows the current recommendation to treat only parasitologically confirmed malaria with ACTs. The impact of post-treatment prophylaxis from case management in situations where long-acting antimalarials are used presumptively may be greater, and the additional benefit of deliberate chemoprevention (whether EPI-IPTi or seasonal IPT) correspondingly lower.

However, an important caveat is that the impact of long-acting drugs predicted by this model is based on the assumption that in a clinical trial, appropriate case management would be given to a high proportion of symptomatic malaria cases. In reality, a smaller proportion of the overall burden malaria cases are likely to be treated and the impact of long-acting drugs on protecting individuals who actually have malaria will be smaller. For this reason, our findings do not support the use of long-acting treatment drugs as an alternative to either IPT strategy, but rather as a complementary approach.

Our results indicate that the use of a long-acting regimen for case management of malaria in conjunction with either form of IPT would be more effective than either form of IPT or long-acting ACTs as a lone strategy. This is biologically plausible given that case-management cannot prevent first episodes, but will prevent additional episodes in highly exposed children, whereas EPI-IPTi or sIPT can also prevent initial malaria episodes. Thus, while providing long-acting drugs for treatment may be of some value where no infrastructure exists to deliver IPT or other forms of chemoprevention (either via the EPI or via other approaches), this will not be as effective as use of long-acting drugs in addition to deliberate chemoprevention by IPT. These findings are in agreement with two studies in Ghana of seasonal IPT in addition to home management of malaria with long-acting drugs [33] and [34].

For most of the range of transmission patterns considered, IPT courses given in infancy during the part of the year when transmission peaks predicted higher efficacy than a four-course EPI-linked IPT program, with a clear advantage in settings with ‘marked seasonality’ [31]. Given that EPI-IPTi is likely to incorporate only three courses (given with DTP2, DPT3 and measles) rather than the four courses included in this simulation, and that seasonal IPT could potentially be optimised further (by including more courses or adjusting the interval to suit the particular drug used), these differences may be conservative. Under uniform perennial transmission, the model predicts that four courses of monthly seasonal-IPT in infancy would give slightly less protection to infants than four courses of conventional EPI-IPTi. However, this could be improved by spacing courses further apart so that the maximum benefit is obtained from each course (i.e. there is no overlap between periods of prophylaxis).

The results presented show that if a delivery system can be developed outside the EPI for the delivery of seasonal IPT, inclusion of young children in addition to infants would offer important advantages. However, the challenges of developing an alternative delivery system could be substantial. In addition to requiring an alternative delivery mechanism, seasonal IPT given for the first few years of life will require more courses of IPT than EPI-IPTi (even if fewer courses are given per year), may achieve lower coverage, and would be more costly. However, the gains achieved by protecting older children who are at high risk of severe malaria could be substantial.

We have limited our analyses to children less than two years old and focused on protective efficacy at 24 months of age for two reasons. Firstly, in order to model the Navrongo cohort in detail, which was only followed-up until two years of age, and secondly, to show the impact on extending for a single year beyond infancy. Our model suggests that with four monthly courses of seasonal IPT in each of the first two years of life, PE would have been 51.7% at 24 months of age. Equivalent protective efficacy to IPT strategies targeting only infants may be achievable with lower coverage or fewer courses. Where seasonal IPT has been evaluated in practice, older children up to 5 or 10 years have been included. The potential advantages of a delivery system capable of administering IPT to older children would be even greater than that shown here. Furthermore, the protective efficacy of seasonal IPT covering a broader age range would be less affected by any future shift in the age-specific disease burden as a consequence of reduced malaria transmission than would EPI-linked IPT given only to infants. In the context of reducing malaria transmission in some parts of Africa [16], and widespread efforts to reduce transmission in other areas, this may be a very important advantage.

Despite the potential advantages of seasonal IPT in children, there are several important factors in addition to protective efficacy that must be considered when comparing seasonal IPT to EPI-IPTi. Several factors were not taken into account in the models, which focused on the direct protection against clinical malaria. This analysis does not consider potential differences in the cost of the alternative delivery strategies, which will weigh against the gains in protection since EPI-IPTi has been shown to be highly cost-effective. However, seasonal IPT using community based health-workers has been piloted in several countries, and can achieve high coverage at moderate cost [35], [36]. Other concerns include the potential impact on drug resistance, possible rebound effects, and the safety of multiple courses. However, the demonstration that equivalent efficacy can be achieved with fewer targeted courses appears to be an advantage of seasonally-targeted delivery in settings in which SP resistance is limited.

Rebound effects were not modelled since the mechanisms by which such effects might be seen are only speculative at present. Nevertheless, this issue requires further study since a temporary increase in incidence of clinical malaria was observed in children given long-term chemoprophylaxis [37]. The effect of SP resistance on IPTi efficacy has been addressed in other studies [38], [39], and the effect of IPT on resistance has also been addressed by modelling studies [40]. These studies found an overall trend of decreasing efficacy with increasing SP resistance, but the exact relationship between prevalence of key resistance markers and IPT efficacy is not well characterised. We considered whether the findings of this study would be robust in settings with different SP resistance patterns by varying curative efficacy and the duration of post-treatment prophylaxis after IPT (the two key aspects that would be affected by SP resistance). While increasing SP resistance does lower the efficacy of each dose of IPT and shorten the period of prophylaxis, leading to lower estimates of protective efficacy, the relative advantage of seasonal IPT over EPI-IPT would remain at a given level of resistance (Supporting Information S1).

Our model was initially parameterised using data collected in Navrongo, Ghana. Some of the values of parameters are likely to differ in other settings, including the proportion of infections that are symptomatic, the recovery rate, and the proportion of infectious bites that lead to a successful infection [41], [42]. The efficacy of IPT strategies and of treatment drugs will also vary in different settings. Despite these limitations, the broad findings of this analysis did not depend strongly on specific values of any parameter. Furthermore, our predictions are consistent with results of trials that have investigated different IPT dosing schedules in seasonal transmission settings [6], [7], [8].

In summary, while IPTi delivered through the EPI is likely to be a valuable malaria control tool in certain situations, particularly those with high transmission, seasonal IPT targeted at the peak transmission period may provide greater protection in other epidemiological settings. Long-acting regimens for case management may complement the protection provided by IPT, but for case-management to have an impact as large as that indicated here, it will be necessary to ensure that these antimalarials reach the children who are most exposed to malaria, typically the individuals who also have poorer access to treatment. Extending seasonal IPT to include young children as well as infants has important additional advantages, and may become increasingly relevant in the future if the reductions in malaria transmission seen in parts of Africa can be achieved more widely and can be sustained. Developing effective delivery mechanisms for these alternative strategies is crucial to ensure the potential advantages presented here can be achieved in practice.

## Supporting Information

Supporting Information S1 **Extended Methods, Results and Sensitivity Analysis.**
(DOCX)
