# Supplementary material for: Modelling the Protective Efficacy of Alternative Delivery Schedules for Intermittent Preventive Treatment of Malaria in Infants and Children
Source: PLoS One. 2011 Apr 20;6(4):e18947. doi: 10.1371/journal.pone.0018947 (PMC3080380; doi:10.1371/journal.pone.0018947)
Supplement: Supporting Information S1 — Extended Methods, Results and Sensitivity Analysis. (DOCX) [file pone.0018947.s001.docx]

**Supporting Information S1. Extended Methods, Results and Sensitivity Analysis**

**Expanded model description**

A set of deterministic compartmental models were developed, which can be expressed as sets of differential equations. For each model, the equations were integrated using the fourth order Runge-Kutta method with a time-step of 0.5 days using Berkeley-Madonna software (version 8). Results of the model were consistent when time-step size was varied between 0.02 and 1 day.

For simplicity, there is no mosquito component in the model; this is replaced with a function fitted to EIR data from the Navrongo site (described in detail below). Implicitly, this approach assumes that the IPT interventions applied to infants do not influence the population-level of transmission. The infection status of the human population is described by a compartmental model developed by a series of extensions to the well-known Ross-MacDonald model of malaria transmission. The key amendments in the basic model are the incorporation of 1) a latent period between infection and development of parasitaemia, 2) superinfection, 3) distinction between symptomatic and asymptomatic parasitaemia, and 4) the effect of treatment. Incorporation of the effect of IPT and seasonal changes in transmission intensity are discussed in a separate section later.

An overview of the basic model structure is shown in figure S1. A description of the meaning of parameters for reference is given in table S1. Values of the parameters and references from the literature related to these are given in table S3. Developments and refinements to the model are explained as appropriate.

**Figure S1. Simplified view of the model structure**

U

E_1_

A

S

E_2_

*λ(t)*

*λ(t)*

θ *f*

(1-θ) *f*

θ *f*

(1-θ)*f*

*r*

q

Overview of different compartments in the model. U, uninfected; E_1_, latent/incubating primary infection; A, asymptomatic infection; E_2_ latent/incubating superinfection; S, symptomatic infection that is treated. Arrows indicate transfers between different states and are described in the text and in table S1.

**Table S1. Description of parameters used in the basic model of IPT (model 1)**

| **Parameter** | **Description / details** |
| --- | --- |
| ε | Enrolment rate |
| α | Ageing |
| EIR | Entomological inoculation rate |
| b | probability an infective bite results in infection in a child |
| *λ* | Force of infection = EIR*b |
| d_e_ | Mean duration of incubation period |
| f | rate infected children progress through incubation period = 1/d_e_ |
| d_a_ | Mean time spent with asymptomatic parasitaemia if untreated |
| r | Recovery rate between each asymptomatic compartment (4/d_a_) |
| θ_0_ | Proportion of new infections symptomatic *and treated* in infants |
| θ_1_ | Proportion of superinfections that are symptomatic *and treated* in 1 year olds |
| d_t_ | Mean duration of prophylaxis after treatment |
| q | Rate of loss of prophylactic protection after treatment (5/d_t_) |
| IPT_cov_ | IPT Coverage |
| IPT_eff_ | IPT Efficacy |
| τ _max_ | Rate individuals should receive IPT (i.e. potential rate if 100% coverage and 100% efficacious):  -EPI: as enrolment + delay (30.4, 60.8, 212.9, 304.2)  -sIPT: pulse at intervals |
| τ | Rate individuals actually receive IPT = τ _max_*IPT_cov_*IPT_eff_ |
| d_IPT_ | Duration of prophylaxis after IPT |
| ψ | Rate of loss of IPT protection (5/d_IPT_) |

This table is provided for reference of the meaning of parameters described in the following section. For values of parameters used in the model and references, see table S3.

In the model children can be in one of the following states: uninfected (U), previously uninfected incubating infection (E_1_), previously asymptomatic incubating superinfection (E­_2_), asymptomatic parasitaemia (A), symptomatic (S).

Children entering the trial are assumed to be uninfected and hence in state U. Children become infected at rate, *λ(t)*, which is the product of the entomological inoculation rate at time (t) and the probability that an infective bite results in infection, *b* (thus *λ(t)* = *b*EIR(t)*). Infected individuals move into E_1_. The incubation period is modelled with an exponential distribution, i.e. the rate individuals develop symptoms or asymptomatic parasitaemia and leave E_1_ is *f=1/d_e_* where *d_e_* is the expected duration of the incubation period.

**Symptomatic and asymptomatic infections**

A proportion, *θ*, of new infections become symptomatic at the end of the incubation period and move into compartment S. The remainder *(1*- *θ)* develop asymptomatic parasitaemia and move into A. For simplicity it is assumed that once asymptomatic, children do not subsequently develop symptoms unless reinfected. The simplified structure shown in figure S1 is developed so that children with asymptomatic parasitaemia recover via three additional compartments (A_2_, A_3_, A_4_) before returning to the susceptible state. Four asymptomatic compartments are used because this makes the distribution of time spent infected more realistic [1]; it also allows a simple means to include the effect of superinfection [2]. Rate of transfer between each compartment is *r=4/d_a_* where *d_a_* is the total expected duration of an untreated infection.

**Superinfection**

Children with asymptomatic parasitaemia can be infected again in the same way as uninfected children. Individuals infected before they recover enter a second incubation period, E_2_, after which a proportion *θ* develop symptoms and are treated (moving from E_2_ into S), or remain asymptomatic and return to A_1_. In the simplest case, *θ*  takes the same value for both new infections and superinfections (i.e. there is no difference in the probability of symptoms when children have an existing infection). This was varied in later versions of the model.

Because the intention was to fit the model to trial data on malaria incidence, it was assumed that all symptomatic children who were detected by trial staff would have been treated. Children who had symptoms but did not report these can be considered to have asymptomatic parasitaemia, at least from the point of view of malaria incidence data collected by the trial team.

**Treatment and post-treatment prophylaxis**

Children who are treated are assumed to recover faster than children who are not treated. Additionally, children given treatment receive a period of chemoprophylaxis when they cannot be infected. This is modelled by delaying the return of treated children to the uninfected (and susceptible) compartment, U, via a series of five sub-compartments, S_i_. The duration spent in each of these is exponentially distributed, and the rate of transfer between each compartment, *q = 5/d_t_* where *d_t_* is the expected total duration protected by treatment. The total duration of protection then follows a gamma distribution rather than an exponential distribution, which is a more plausible distribution because the mode will not be zero.

Levels of drugs are not modelled explicitly. However, the pattern of waning protection from malaria is described by the profile of the curve describing duration of protection, which captures the fact that protection will be lower later in the prophylactic period (i.e. the majority of children given treatment will no longer be protected). To model the levels of drugs explicitly, one would need to simulate the sensitivity thresholds of different circulating parasites and then model drug levels in individuals, rather than the compartmental structure. The approach chosen is for simplicity, and because there is insufficient evidence of what concentration of specific drugs provides protection in vivo.

Chloroquine (CQ) was the main antimalarial treatment used during the Navrongo trial. While prophylaxis times may be substantial for sensitive parasites, CQ was failing in KND at the time of the study (14 day adequate clinical and parasitological response 47% [3]). Post-treatment prophylaxis against erythrocytic stages may last for as little as two weeks where CQ resistance is high [4].

**The basic model excluding IPT**

A diagram of the basic model structure incorporating superinfection and prophylaxis after treatment is shown in figure S2. The differential equations describing movements between the compartments of the model are shown below. Incorporation of IPT and age group is discussed in the next section.

**Equations for the basic model excluding IPT**

**Number of children under observation and initial conditions**

Because the number of children are modelled (rather than the prevalence in all children) the sum of the different states in the model is the total number of children currently under observation. All states of the model begin with the value zero. Expressed mathematically,

**Force of infection**

The force of infection acting on children depends on the entomological inoculation rate, *EIR*, (the number of potentially infective bites per human per unit time), and the proportion of infective bites that are infectious to humans, *b*. The EIR is either modelled as a function fitted to entomological data, or a hypothetical function to simulate the EIR in a range of transmission patterns. Further details are given below in the section ‘Fitting the entomological inoculation rate’

at time *t*, $\lambda(t)=EIR(t)*b$

**Figure S2. Diagram of the basic model excluding IPT**

U

E_1_

A_1_

S_1_

A_2_

A_3_

A_4_

E_2_

*λ(t)*

θ *f*

(1-θ)*f*

θ *f*

(1-θ)*f*

*r*

*r*

*r*

*r*

q

S_5_

S_4_

S_3_

S_2_

q

q

q

q

*λ(t)*

*λ(t)*

*λ(t)*

*λ(t)*

Compartments and transfers in the basic transmission model. U, uninfected; E_1_, latent/incubating primary infection; A_1_-A_4_ asymptomatic infection; E_2_ latent/incubating superinfection; S_1_-S_5_, symptomatic infection that is treated and protected against subsequent infections by prophylaxis. Arrows indicate transfers between different states and are described in the text.

**Incorporation of IPT into the basic model**

The model described above describes the transmission dynamics common to all participants in the Navrongo trial. Incorporation of intermittent preventive treatment to an otherwise identical model represents the group of infants receiving IPT. The two models are then run in parallel to allow simulation of the treated and untreated trial cohorts. Two aspects of the mode of action of IPT are dealt with by this model, the curative aspect and the prophylactic aspect.

It is assumed that an effective dose of IPT will clear existing parasitaemia and will clear any infections already incubating at the time of receiving IPT once they emerge from the liver. Individuals given an ineffective dose of IPT remain as if they had not been treated at all. In addition to its curative effect, IPT is considered to provide prophylactic protection in individuals who are successfully treated. The concentration of drug required to clear parasitaemia will depend on the susceptibility of the parasite itself and the anti-parasite mechanisms of the host which act in synergy with the drug [5,6]. This variation results in a protection over time following an approximately sigmoid shape.

Infants successfully treated with IPT move from all other compartments in the model into the IPT component of the model and remain in this state for a variable period before returning to U. As for the treatment component, the IPT component is modelled with a gamma distribution rather than an exponential distribution, obtained by subdividing the state into five successive compartments; this component then provided a distribution very similar to that observed in the analysis of duration in [7]. The rate of transfer between each IPT compartment is *ψ =* *5/d_IPT_* where *d_IPT_* is the expected total duration of protection from IPT. More detail on this is given later in the section on fitting the model (figure S6).

**Inclusion of IPT dosing strategies**

IPT administration is modelled in one of two ways, reflecting the two basic strategies of EPI-linked IPT and seasonal-IPT. The rate at which individuals scheduled to receive IPT actually enter the protected state, *τ*, depends on the coverage and efficacy of IPT (denoted *IPT_cov_* and *IPT_eff_* respectively) in addition to the delivery schedule. Implicitly, it is assumed that coverage of each dose is independent of the previous dose. This may be slightly different to what occurs in reality: individuals who miss one course may be more likely to miss a subsequent course [8]. Reasons for IPT efficacy less than 100% are not distinguished separately but are considered to include infection with a drug resistant parasite, insufficient or incorrect dose, vomiting after the dose, etc.

**1) EPI-linked IPT**

The number of children scheduled to receive IPT follows the ageing pattern of the cohort after enrolment at two months of age. The number scheduled to receive IPT is the same as the number enrolled plus a delay of one month (30.4 days) for IPT-1 (given at three months of age) , two months (60.8 days) for IPT-2 (given at four months), seven months (212.9 days) for IPT-3 (given at nine months) and ten months (304.2 days) for IPT-4 (given at 12 months of age). Implicitly, it is therefore assumed that children receive vaccinations at the scheduled times.

**2) Seasonally targeted IPT**

Infants are scheduled to receive IPT at defined time points in the year. When IPT occurs depends on the dosing schedule chosen: the time the first dose is given each year, the number of IPT rounds per year and the interval between each round.

**Age structured model incorporating IPT in infants (model 1)**

For simplicity, the model does not take detailed account of changes in age. However, children over 1 year of age would no longer receive IPT if IPT is targeted only at infants. Incidence is known to vary with age, but in practice the differences appear small when compared to the temporal differences over the course of the year (figure 3 in main document). To capture these differences, the probability of symptoms upon infection is allowed to vary between age groups: θ is denoted θ_0_ for infants and θ*_1_* for one year old children.

For the age-structured model both the SP and placebo groups are split into two age strata, representing infants and one year old children. The model thus has four human components, all identical in structure except for IPT compartments in the SP group. One year old children retain the IPT compartment because children can reach one year of age while still protected by IPT even if IPT is not given after infancy.

**Simulation of enrolment and ageing**

Time in the model represents calendar time from the beginning of the trial in 2000 until its completion in 2004. Model time begins on 1^st^ September 2000. Because enrolment started at the end of September 2000, there is a run in period of 30 days before enrolment begins in the model. This run in was allowed so that EIR could be modelled from September 2000 onwards. The initial condition of each model compartment is therefore zero.

The number of study children who were born, enrolled and had exited over the course of the study is shown in figure S3. The enrolment function in the model follows this pattern, with a constant rate of enrolment between day 30 (1^st^ October 2000) and day 668 (1^st^ July 02). The model slightly overestimates the number of children remaining in the trial because it does not account for early exit or death. Because children were enrolled at two months of age, individuals transfer to the one year old stratum after spending 305 days (10 months) in the infant stratum. The function for ageing into the one-year old stratum is thus identical to the enrolment function with a delay of 305 days (10 months). Children exit the one year age group (and thus the entire model) 365 days after entering it, i.e. 670 days after first entering the infant stratum.

**Figure S3. Birth, enrolment and exit patterns for the IPTi trial and the model**

Figure shows patterns of birth, enrolment and exit from the IPTi trial dataset. The model approximation of these patterns is also shown.

**Mathematical notation for time dependent parameters**

Most parameters in the model remain the same throughout each model run. Those that vary with time, denoted t, are as follows:

- the enrolment rate of children (children per day) enrolled into the trial cohort, ε(t)
- the ageing of children, α(t), which is equal to the enrolment function with a fixed delay of 305 days for infants and 670 days (305+365) for one year old children.
- the rate at which individuals are treated with IPT, denoted τ(t)
- the EIR and the parameters which depend on it (λ(t)). Changes in EIR and λ(t) with time are described in more detail in the sections on ‘Extensions to the basic model’ and ‘Fitting the model’, below and are not discussed further here.

**Enrolment of infants and ageing**

Let a denote the age-group. a=0 denotes infants (2-12 months of age) and a=1 denotes one year old children (12-23 months of age). a’ is the complement of a (i.e. if a=0 then a’=1 and if a=1 then a’=0).

Let ε denote the enrolment rate.

ε(a,t) = ε(t) if a=0, and

0 otherwise.

ε(t)=0 (t<30); ε (t)=1.95 per day (30<=t<=668); ε (t)=0,t>668

To model age group, a number of individuals equal to the number enrolled 305 days previously move from age group 0 to age group 1. The number from each compartment of the model moving from age group 0 to the corresponding compartment in age group 1 is calculated to give a total per unit of time equal to the number enrolled 305 days previously:

α(a,t) = - α(t) if a=0 , and

α(t) if a=1.

Let j denote all the compartments of the model in age group a.

α(a,j,t)= ε(t-305)*n(a,j,t)/N(a,j,t) where n(a,j,t) is the number of individuals in compartment j in age group a at time t, so the total sum of α over all compartments in age group 0 is equal to ε(t-305). Ageing in age group 1 is analogous but equal to ε(t-670).

**Treatment**

Let z denote the intervention group. z=0 denotes placebo and z=1 denotes SP. Then:

τ(t,z) = 0 if z=0, and

τ(t) if z=1

τ varies according to the particular IPT strategy used. This is described in detail elsewhere.

The differential equations describing movements between the compartments of the model are shown below. A diagram of the entire basic transmission model is shown figure S4. As before, the sum of all the states gives the total number of children under observation in each combination of intervention group and age group. At time t=0, the number of children in all compartments, denoted N(a,z)=0.

**Equations of the basic model incorporating IPT and age group (model 1)**

For clarity of presentation only the time-dependent parameters are denoted by t. Implicitly, states of the model e.g. U(a,z), E_1_(a,z), etc. are also time dependent, but following convention the notation for time-dependency of states is omitted.

**Figure S4. Age-structured model incorporating IPT in infants (model 1)**

U

E_1_

A

S

E_2_

IPT

**Placebo group**

**SP group**

**Infants**

**One year olds**

*α(t)*

U

E_1_

A

S

E_2_

IPT

U

E_1_

A

S

E_2_

U

E_1_

A

S

E_2_

*τ(t)*

ψ

ψ

*α(t)*

*α(t)*

*α(t)*

*τ(t)*

*τ(t)*

*τ(t)*

*τ(t)*

Ageing applies equally to all compartments in the model. For clarity of presentation, parameters except for those involving IPT (*τ(t)* and *ψ*) and ageing (*α(t)*) are omitted and multi-compartment components for asymptomatic infection, treatment and IPT are shown as a single compartment. Full details are given in the equations that follow. The one year old SP group has an IPT component because children in the IPT compartment who reach one year of age may still be protected from a dose received during infancy. There is no flow into the IPT compartment in the one year age group because it is assumed that the latest a dose of IPT can be given is just before 12 months of age.

**Extensions to the basic model**

Two extensions are made to the full model to incorporate the effect of maternal immunity and heterogeneity in malaria exposure. Additional parameters of the extensions are described in table S2.

**Table S2. Additional parameters used in extensions to the basic model**

| Parameter | Description |
| --- | --- |
|  | Maternal immunity (model 2 and model 3) |
| d_m_ | Duration of maternal protection after enrolment at 2 months of age |
| γ | Rate of loss of maternal immunity in each compartment (4/d_m_) |
| p_maternal_ | Probability that an infective bite that results in successful infection *(λ*b)* succeeds in a child protected by maternal immunity |
| *λ_m_* | Force of infection acting on maternally protected children |
|  | Heterogeneity (model 3) |
| *λ(x)* | Force of infection according to exposure group. For less exposed children x=0; for highly exposed children x=1 |

**Maternal immunity (model 2)**

Maternal immunity was modelled by adding an additional set of protective compartments: children enter the model into a series of four compartments (M_1_ - M_4_) representing a protected state. Waning of immunity is denoted by *γ=4/d_m_*_,_ where *d_m_* is the mean total duration of maternal protection. Transfer between each compartment is thus exponential and individuals move from last the maternally protected component into the uninfected compartment, U, following a gamma distribution. Children protected by maternal immunity are exposed to the same EIR and can still be infected, but the probability of a successful infection is much lower, denoted by a modified force of infection *λ*_m_ = *λ**p_maternal_ where p_maternal_ represents the proportion of successful infections that overcome maternal protection. Biologically it may be the case that maternal protection is against symptoms rather than infection *per se* [9], this approach is chosen for simplicity. A diagram of the maternal immunity component is shown in figure S5.

Because children were not enrolled into the IPTi trial until two months of age it is unclear how important the remaining maternal protection is. For this reason the effect of its inclusion was examined by comparison with model 1. This is consistent with the approach of other IPTi modelling studies e.g. Gosling et al. [10].

**Figure S5. Incorporation of the maternal immunity component**

U

E_1_

enrolment

enrolment

**No maternal protection**

**With maternal protection**

*f* θ_1_

*f*(1-θ_1_)

M_1_

M_2_

M_3_

M_4_

E_1_

*λ_m_(t)*

*4/d_m_*

U

*4/d_m_*

*4/d_m_*

*4/d_m_*

*f* θ

*f*(1-θ)

*λ(t)*

*λ(t)*

*λ_m_(t)*

*λ_m_(t)*

*λ_m_(t)*

**Equations of the model incorporating maternal immunity (model 2)**

**Heterogeneity (model 3)**

Malaria infections are overdispersed, with a few individuals suffering a disproportionate proportion of the disease burden.. On average (over several studies) approximately 20% of the population were found to bear 80% of all infections, while the remaining 80% of individuals experienced only 20% [11]. The effect of heterogeneity on the efficacy of different IPT strategies was investigated by creating a human model with two levels of exposure, one representing 20% of the trial cohort who were highly exposed and the other the remaining 80% with low exposure. Only the force of infection varies between the levels. The different levels of the model are given the additional index *x*: 0 represents the less exposed group and 1 the highly exposed group. The notation for the model incorporating heterogeneity is thus a,x,z where a denotes age, x denotes exposure and z denotes intervention group. Implicitly, this assumes that the degree of exposure is independent of access to IPT and treatment, although this can be varied in the model. The impact of high exposure being correlated with poor access to IPT is explored later.

In order that the infection burden divides with 80% of infections occurring to 20% of the population and 20% to 80% the force of infection is modified. This is denoted *λ(x)*, where x=0 denotes the less exposed group and x=1 the highly exposed group. Children in the highly exposed group receive four times the average recorded daily EIR; children in the lower exposure category receive one quarter of the average daily EIR. This simple scaling of force of infection is chosen for simplicity and ignores additional complications that would occur in reality e.g. that highly exposed children might be at risk of infection throughout a longer period of the year than children with lower exposure.

**Equations of the model incorporating heterogeneity (model 3)**

**Fitting the models**

Fitting the components which describe the duration of IPT protection and the entomological inoculation rate was done first before incorporation into the full model.

**Fitting the duration of IPT protection**

Since protection after IPT is modelled by delaying return to the uninfected susceptible state (i.e. to a state in which a child can receive an infection that will progress normally) it is necessary to subtract the prepatent period from the time spent protected. A new inoculation received approximately one week before drugs fall below parasiticidal levels may succeed because the liver stage will last between 5-7 days for *P. falciparum* [12], during which time SP does not kill the parasites. The duration spent in the protected (I_i_) compartments of the model before return to the U compartment is therefore estimated as being between 7-11 days shorter than the observed protection against clinical malaria. This is based on one prepatent period of 5-7 days plus 2-4 days for parasites to complete one or two 48 hour life-cycles to increase parasite density and cause symptoms.

The protective efficacy against clinical malaria approximately follows a gamma distribution with a mean 35 - 40 days (figure S6). Discounting 7-11 days suggests that the duration spent in the protected compartments of the model should be modelled with a gamma distribution with a mean of 30 days. 35 days appears likely to be a slight overestimate and 20 days slightly conservative.

Analogously, the minimum 14 days of prophylaxis against blood-stage infection with resistant parasites after CQ treatment [4] is modelled with a minimum of 7 days spent in the treatment component to allow for the lag between inoculation and patency.

**Figure S6. Duration of protection after IPT**

Proportion of those treated with IPT remaining in the protected compartments of the model (delay before returning to U compartment) by time, with total mean duration of protection set at 20, 30, 35 and 40 days. Protective efficacy of SP against clinical malaria from Cairns et al. [7] is also shown.

**Fitting the entomological inoculation rate**

Data on the entomological inoculation rate for the Kassena-Nankana district between November 2001 – October 2004 were used (Anto et al. [13], unpublished). A function describing the log EIR was used to model seasonality. The functions were Fourier series of the form:

The term a_0_/2 is the the mean value of the function, and a_n_ and b_n_ are a series of fourier coefficients. The parameters a_0_, a_1…n_, b_1…n_, N and T were allowed to vary in order to provide the best fit to the EIR data for each year. Fits to data from each of the four years were performed separately using least squares in Berkeley-Madonna. The fitted functions for EIR in each year were then combined together to give a smooth function for the EIR during the entire study period (figure S7).

**Figure S7. Entomological inoculation rate during the IPTi trial**

Points show data on estimated EIR by Anto et al (unpublished data). The line shows the EIR function fitted in the model.

**Fitting the models to malaria incidence data**

All three models were fitted to the data using the approach described below. Malaria incidence data from the Navrongo trial was used for the final fit of the models. For all three models, malaria incidence by the end of each month in the model was fitted to monthly cumulative incidence data from the Navrongo trial cohort. Cumulative malaria incidence in the model was fitted simultaneously to incidence data from the four groups enrolled in the trial (infants and one year old children in the SP and placebo groups). Fitting was carried out by varying parameters in the model in order to maximise the binomial log-likelihood function, using the optimize algorithm in Berkeley Madonna. Model parameters and ranges are described in table S3.

The log-likelihood was calculated for each month as:

where *x* is the number of malaria cases (in the data), *n* is the number of children at risk (in the data) and *p* is the risk in that month (predicted by the model). The binomial log likelihood was used as a measure of goodness of fit. The total log-likelihood for a particular model fit was calculated by summing across months. If *c* indicates calendar month, then the total likelihood is

where for each calendar month bounded by the interval t1 and t2

Model parameters were varied within the ranges specified by the literature review to determine the best fitting and most plausible description of malaria incidence in the Navrongo trial site. Starting values for parameters was based on the literature review. Due to the large number of parameters in the model, most model parameters were fixed for the final stages of fitting the model to malaria incidence data. However, all parameters were within the plausible ranges indicated by the literature review and for several parameters it was possible to use the values from studies undertaken in the area (Navrongo or Kassena-Nankana District) around the time of trial. The final values and the range considered are shown in table S3. The two parameters used for the final stages of fitting are described below.

**Parameters used to fit the final model to malaria incidence data**

The parameters that have most influence on the incidence of malaria predicted by this model and that were used to fit the final model to malaria incidence data were 1) the proportion of infective bites that result in human infection, *b*, and 2) the proportion of infections that were symptomatic and and treated, *θ*. The values of *b* and *θ* are not certain and were therefore estimated by allowing them to vary within a prespecified range (0.005-0.1 and 0.05-0.35 respectively, table S3). Allowing *θ* to take a different value for superinfections (compared to first infections) did not improve the fit of the model to the data, and in some cases resulted in implausible values being predicted. Consequently, the probability of developing symptoms upon superinfection is assumed to be the same as upon first infection.

The full model incorporating maternal protection and heterogeneity in transmission (model 3) was best fitting. Log-likelihood values were used for the purpose of fitting the models to the data rather than for formal statistical comparison of the different models. The incorporation of maternal immunity and heterogeneity did improve the fit of models, but even if they had not done so it would still have been of interest to explore the possible impact of protection early in life and heterogeneity. Model 3 was used for making the predictions presented below. Discussion of results from the simpler models is given as appropriate.

**IPT coverage and efficacy**

Adequate clinical and parasitological response 14 days after treatment with SP for symptomatic malaria in this area was estimated as 77.6% (95% CI: 68.9, 84.8) [3]. Antimalarials may be more efficacious at clearing new infections than existing ones, which typically have higher parasite densities [14]. Consequently the efficacy of SP when used for IPTi could be slightly greater than that for treatment of symptomatic malaria. The fit of the model was best when the assumed efficacy of SP was 85-90% (85% for models 1 and 2 and 90% for model 3). This means that 90% of children given IPT are assumed to clear an infection (if they have an infection at the time of IPT), and enter the protected compartments of the model. This level of efficacy seems reasonable given that approximately a quarter of children in the placebo group were infected at IPT-3 and IPT-4 in Navrongo [15]. Assuming 22% of these experienced treatment failure when given SP for IPTi one would still expect less than 6% of all children to experience recrudescence after IPT. In other words, assuming an efficacy of 90%, as used in the model, does not imply an unrealistically high efficacy of SP, and allows for a small amount of reduction in efficacy due to reasons apart from drug resistance (inadequate doses, vomiting after treatment, etc.).

**Table S3. Descriptions and values of parameters used in the final model**

| **Parameter** | **Description / details** | **Values from literature / data** | **Citation / source of estimate** | **Best estimate** | **Range** |
| --- | --- | --- | --- | --- | --- |
| ε(t) | Enrolment rate | Varies with time: 1.95/arm/day between days 30 and days 668 | Navrongo data | 1.95 per arm | - |
| α(t) | Ageing  infants to 1 year olds | Varies with time:  Enrolment rate + delay of 305 days | - | - | - |
|  | 1 year old to exit from model | Enrolment rate + delay of 670 days | - | - | - |
| EIR | Entomological inoculation rate | Varies with time according to data (Navrongo) or sinusoidal function (other settings) | Anto et al., unpublished | See description | - |
| b | probability an infective bite results in infection in a child | 0.006 (High EIR), 0.07 (Low EIR) | [16] | 0.02 | 0.005 – 0.1 |
|  |  | 0.026 - 0.073 (EIR=1 per day) | [17] |  |  |
|  |  | 0.03-0.13 | [18] |  |  |
|  |  | 0.1 | [19] |  |  |
| λ(t) | Force of infection = EIR*b | Varies with time: determined by EIR | - | - | - |
| d_e_ | Mean duration of incubation  period | 12 (9-14) days | [12] | 10 days | 7-14  days |
|  |  | 9 days | [20] |  |  |
| f | rate infected children progress through incubation period  = 1/d_e_ |  | - | - | - |
| d_a_ | Mean time spent with  asymptomatic parasitaemia  if untreated | 23 days (highly endemic) | [21] | 100 days | 50-200 days |
|  |  | 73 days < 18 months  160 days 18 – 23 months | [22] |  |  |
|  |  | 210 days (176-256) | [23] |  |  |
|  |  | 210 days (183-236) | [24] |  |  |
| r | Recovery rate between each asymptomatic compartment =(4/d_a_) |  | - |  |  |
|  | Proportion of new infections that are symptomatic | 0.31 (KND, dry season),  0.34 (KND, wet season) | [25] |  |  |
|  |  | 0.44 (Kenya) | [26] |  |  |
| θ(0) | % infections symptomatic *and treated* in infants (a=0) |  | Parameter fitted by the model | 23.7% | (0.05 – 0.35) |
| θ(1) | % infections symptomatic *and treated* in 1 year olds (a=1) |  | Parameter fitted by the model | 20.4% | (0.05 – 0.35) |
| d_t_ | Mean duration of prophylaxis after treatment for malaria | 14-day ACPR 47%  14 days PTP after CQ treatment | [3]  [4] | 14 days | 7-21 days |
| q | Rate of loss of prophylactic protection after treatment (5/d_t_) |  |  |  |  |
| IPT_cov_ | IPT Coverage | 0.92 | Navrongo data | 0.92 | (0.5-0.95) |
| IPT_eff_ | IPT Efficacy | 14 day ACPR: 0.78 | [3] | 0.9 | (0.5-0.95) |
| τ _max_ | Theoretical maximum rate individuals could receive IPT protection if coverage and efficacy both 100% | EPI: as enrolment + delay  sIPT: pulse at intervals |  |  |  |
| τ(t) | Rate individuals move into protected compartments after IPT = τ _max_*IPT_cov_*IPT_eff_ | Varies with time according to IPT strategy | **-** |  |  |
| d_IPT_ | Duration of prophylaxis after IPT | 4-6 weeks (SP) | [7], Gamma distribution fitted to data | mean 30 days | 20-35 days |
|  |  | 60 days maximum | [5] |  |  |
| ψ | Rate of loss of IPT protection (5/d_IPT_) |  |  |  |  |
|  |  |  |  |  |  |
|  | Maternal protection |  |  |  |  |
| d_m_ | Duration of maternal protection after enrolment at two months | Protected up until 4 months of age | [9]  [27]  Estimated mean of gamma distribution | 30 days | 10-60 days |
| γ | Rate of loss of maternal immunity in each compartment (4/d_m)_ |  |  |  |  |
| p_maternal_ | Probability that an infective bite that results in successful infection *(λ*b)* succeeds in a child protected by maternal immunity | 0.05 (estimated from [9], no specific value given) |  | 0.05 | 0.001 – 0.0001 |
| *λ_m_* | Force of infection acting on maternally protected children | *λ*p_maternal_* |  |  |  |
|  |  |  |  |  |  |
|  | Heterogeneity |  |  |  |  |
| *λ(0)* | EIR for less exposed children | EIR/4 | [11] |  |  |
| *λ­(1)* | EIR for highly exposed children | EIR*4 |  |  |  |

**Comparison of observed incidence to predictions from the fitted model**

Malaria incidence in each month from the Navrongo trial data and the incidence predicted by the model for the corresponding month is shown in figures S8 and S9. Cumulative incidence over the entire study period and the corresponding model prediction is shown in figure S10. The model predictions were very close to the patterns observed in reality. In particular, predicted cumulative incidence in infancy fitted the observed patterns very closely. The model slightly overestimated cumulative incidence in one year old children, predicting around 100 more cases than occurred in reality, but this was similar in both the intervention and control groups.

**Protective efficacy predicted by the model**

The fitted model predicted protective efficacy for EPI-linked IPT of 25.3% at 15 months of age and 15.6% by 24 months of age. This is very similar to that obtained in practice in the clinical trial (24.9% at 15 months and 16.3% at 24 months of age [15]).

**Figure S8. Cumulative malaria incidence in infants by calendar month**

**Placebo group**

**SP group**

**Figure S9. Cumulative malaria incidence in one year old children by calendar month**

**Placebo group**

**SP group**

**Figure S10. Cumulative malaria incidence over the entire study period**

**Infants**

**1 year old children**

Cumulative malaria incidence over the entire IPTi trial period is shown for infants and one year old children. Points indicate data from the IPTi trial, lines are the cumulative incidence in the model.

**Sensitivity analysis**

The effect of varying the parameters in the model within the defined range on the protective efficacy of IPTi and seasonal-IPT in infancy is shown in table S4. Varying the parameter values did affect the point estimates of protective efficacy: at 24 months of age IPTi varied between 9.3% and 17.8%; sIPT at 24 months of age varied between 14.8% and 28.6%%. The relative differences between seasonal IPT and IPTi remained similar as parameters were varied. Seasonal IPT was consistently between 1.5 and 1.8 times more protective than IPTi.

**Table S4. Sensitivity of the model to parameter estimates**

|  |  | **Protective efficacy of IPTi (%) at age** | | | **Protective efficacy of seasonal IPT (%) at age** | | | **Relative Protective efficacy at age** | | |
| --- | --- | --- | --- | --- | --- | --- | --- | --- | --- | --- |
| **Parameter** | **value** | **12 m** | **15 m** | **24 m** | **12 m** | **15 m** | **24 m** | **12 m** | **15 m** | **24 m** |
| Lower extent of range | |  |  |  |  |  |  |  |  |  |
| b | 0.005 | 30.9 | 25.3 | 15.0 | 55.9 | 46.0 | 27.3 | 1.81 | 1.82 | 1.82 |
| d_e_ | 7 | 29.4 | 24.2 | 15.2 | 50.0 | 42.0 | 25.9 | 1.70 | 1.74 | 1.70 |
| d_t_ | 7 | 30.7 | 25.7 | 15.9 | 51.4 | 43.3 | 26.8 | 1.67 | 1.68 | 1.69 |
| d_a_ | 50 | 30.2 | 25.3 | 15.6 | 50.3 | 42.3 | 26.1 | 1.67 | 1.67 | 1.67 |
| IPT_eff_ | 0.5 | 17.9 | 14.8 | 9.3 | 29.4 | 24.5 | 14.8 | 1.64 | 1.66 | 1.59 |
| d_IPT_ | 20 | 22.3 | 18.3 | 11.1 | 39.8 | 32.6 | 19.9 | 1.78 | 1.78 | 1.79 |
| d_m_ | 10 | 30.2 | 25.3 | 15.6 | 49.6 | 41.7 | 25.8 | 1.64 | 1.65 | 1.65 |
| p_maternal_ | 0.001 | 30.2 | 25.3 | 15.6 | 50.4 | 42.4 | 26.2 | 1.67 | 1.68 | 1.68 |
| Upper extent of range | |  |  |  |  |  |  |  |  |  |
| b | 0.1 | 29.7 | 24.8 | 15.3 | 44.9 | 37.8 | 23.2 | 1.51 | 1.52 | 1.52 |
| d_e_ | 14 | 31.6 | 26.5 | 16.4 | 50.6 | 42.8 | 26.5 | 1.60 | 1.62 | 1.62 |
| d_t_ | 21 | 29.5 | 24.6 | 15.2 | 49.1 | 41.2 | 25.4 | 1.66 | 1.67 | 1.67 |
| d_a_ | 200 | 30.2 | 25.3 | 15.6 | 50.3 | 42.3 | 26.1 | 1.67 | 1.67 | 1.67 |
| IPT_eff_ | 0.95 | 31.6 | 26.5 | 16.4 | 53.1 | 44.7 | 27.7 | 1.68 | 1.69 | 1.69 |
| d_IPT_ | 35 | 33.8 | 28.6 | 17.8 | 54.0 | 46.0 | 28.6 | 1.60 | 1.61 | 1.61 |
| d_m_ | 60 | 28.6 | 24.0 | 14.8 | 50.1 | 42.1 | 25.9 | 1.75 | 1.75 | 1.75 |
| p_maternal_ | 0.01 | 30.2 | 25.2 | 15.6 | 50.2 | 42.2 | 26.1 | 1.66 | 1.67 | 1.67 |

The table shows the impact of varying parameters between their lowest and highest value (as defined in table S3). 12 m = 12 months, 15 m = 15 months, etc.

**Supplemental analyses and results**

**Effect of coverage on protective efficacy in Navrongo**

Four doses of monthly seasonal IPT with coverage of 53% or greater would be more efficacious than four doses of IPTi with the same coverage as in the clinical trial (92%). Seasonal IPT with coverage of only 46% of would be as protective as IPTi given with likely estimates of EPI coverage (79.5% for Ghana in 2003 [28]).

Coverage might vary in the different exposure groups due to possibility that malaria risk and access to healthcare are correlated. The ability of IPT to target the most exposed cohort of children has a substantial impact on efficacy (table S5 and figure S11). If IPT completely fails to protect the most exposed group (the worst case scenario), efficacy could fall by almost 50% for IPTi relative to the protection if coverage is equal in both exposure groups. Failure to target the most exposed group would also reduce the protective efficacy for sIPT by approximately a third. In the best case scenario, complete coverage of the most exposed group could increase the protective efficacy of both approaches by around one third. The results shown below indicate that four monthly doses of seasonal IPT would only be less protective than IPTi if IPTi was highly successful at targeting the highly exposed group and seasonal IPT was extremely poor at targeting this group. It is unlikely that such large differences would occur in reality.

**Table S5. Effect of coverage on protective efficacy of IPTi and seasonal IPT in infancy**

| Scenario | % coverage by exposure group | | Protective efficacy at 24 months of age (%) | | Relative protective efficacy * | |
| --- | --- | --- | --- | --- | --- | --- |
|  | Low | High | IPTi | sIPT | IPTi | sIPT |
| Equal coverage in both exposure groups | 92 | 92 | 15.6 | 26.1 | - | - |
|  | 90 | 90 | 15.3 | 25.6 | - | - |
|  | 80 | 80 | 13.8 | 23.0 | - | - |
|  | 70 | 70 | 12.3 | 20.3 | - | - |
|  | 60 | 60 | 10.7 | 17.7 | - | - |
|  | 50 | 50 | 9.1 | 14.9 | - | - |
| Best case | 90 | 100 | 16.2 | 27.1 | 1.04 | 1.04 |
|  | 87.5 | 100 | 15.9 | 26.7 | 1.04 | 1.04 |
|  | 75 | 100 | 15.1 | 25.1 | 1.09 | 1.09 |
|  | 62.5 | 100 | 14.2 | 23.4 | 1.15 | 1.15 |
|  | 50 | 100 | 13.3 | 21.6 | 1.24 | 1.22 |
|  | 37.5 | 100 | 12.3 | 19.8 | 1.35 | 1.33 |
| Worst | 100 | 60 | 13.3 | 22.9 | 0.85 | 0.88 |
| case | 100 | 50 | 12.6 | 21.8 | 0.82 | 0.85 |
|  | 100 | 0 | 7.7 | 14.9 | 0.56 | 0.65 |
|  | 87.5 | 0 | 6.9 | 13.1 | 0.56 | 0.65 |
|  | 75 | 0 | 6.0 | 11.4 | 0.56 | 0.64 |
|  | 62.5 | 0 | 5.1 | 9.7 | 0.56 | 0.65 |

* relative protective efficacy compared to scenario with equal coverage of children of low and high exposure status.

**Figure S11. Effect of coverage on protective efficacy of IPT**

The figure shows effect of reducing levels of coverage for IPTi and four doses of seasonal IPT from the levels seen in the IPTi trial (92%). Three scenarios are shown. ‘Equal’ indicates that coverage remains the same in both groups as coverage decreases. ‘Best case’ indicates the efficacy if coverage remains at 100% for highly exposed children despite reduced coverage overall. ‘Worst case’ indicates efficacy if coverage falls first in the 20% of children who are highly exposed, hence the steep decline in efficacy until coverage falls to 80%.

**Effect of long-acting drugs for IPT**

Seasonal IPT would remain more efficacious than IPTi even if the drug used for IPT is longer-acting (table S6). However, the benefits of seasonally-targeted treatment become smaller. With a drug which lasts twice as long as SP, the best four-dose seasonal IPT strategy would still be almost half again as effective. For a drug with a mean duration of protection of 90 days (three times as long as SP and longer than any regimens currently available) there is still a clear improvement from using seasonally targeted IPT.

**Table S6. Protective efficacy of IPTi and sIPT with longer acting IPT drugs**

| Mean duration of IPT protection | Scenario | PE at 24 months of age | Relative PE |
| --- | --- | --- | --- |
| 30 days | IPTi | 15.6 | - |
|  | sIPT optimum start time | 20.9 | 1.34 |
|  | sIPT optimum start & interval | 26.6 | 1.7 |
| 60 days | IPTi | 26.8 | - |
|  | sIPT optimum start time | 36.4 | 1.36 |
|  | sIPT optimum start & interval | 38.6 | 1.44 |
| 90 days | IPTi | 36.1 | - |
|  | sIPT optimum start time | 42.3 | 1.18 |
|  | sIPT optimum start & interval | 45.5 | 1.27 |

Protective efficacy is shown for four doses of IPTi in infancy or four doses of seasonal IPT in infancy alone. For seasonal IPT, either the optimal start time alone is used, or the optimal start time and the optimal interval for four doses

**EIR functions used to extend the model to other sites**

Simple sinusoidal EIR functions were used to simulate varying degrees of seasonality along this scale. EIR functions for 10% intervals (on the scale from 50% to 100% of transmission within 6 months) were created for two different transmission intensities, high (EIR=365, i.e. one infectious bite per individual per night, a similar intensity to Navrongo) and low-moderate (EIR = 10, the lower transmission intensity threshold suggested by WHO for implementation of IPTi). The suitability of IPTi and sIPT were compared under these transmission intensities. For this comparison, IPTi was modeled as it was implemented in Navrongo (4 doses of IPT given at 3, 4, 9 and 12 months of age) and seasonal IPT was modeled as four monthly doses centred around the peak in transmission.

**Figure S12. Variable seasonality patterns: high transmission (EIR = 365)**

80%

70%

60%

50%

100%

90%

Lines show the number of infectious bites per person per night. Large numerical values indicate the proportion of infections that occur in the six months of the year with highest malaria transmission for each graph

**Figure S13. Variable seasonality patterns: low-moderate transmission (EIR = 10)**

80%

70%

60%

50%

100%

90%

Lines show the number of infectious bites per person per night. Large numerical values indicate the proportion of infections that occur in the six months of the year with highest malaria transmission for each graph

**Sites with extremely seasonal transmission**

Some sites, such as the Sahel, have almost all malaria transmission occurring in only a few months of the year. An example is shown for Niakhar, Senegal, where almost all transmission occurs over three months and the total EIR is approximately ten infectious bites per person per year [29] (figure S14). A plausible description of the EIR in the absence of data points was created by using the sin wave parameters a = -62.71, b= 62.87, c= 950, d= 4000 and using a delay function to simulate transmission in latter years.

**Figure S14. Model approximation of entomological inoculation rate in Niakhar, Senegal**

Figure shows daily and total EIR for simulation of an extremely seasonal transmission setting with a total yearly EIR of 10.

Giving a single dose of seasonal IPT to infants alone at the peak of transmission is superior to 4 doses of IPTi through the EPI all year round. Seasonal IPT given once in infancy and once in the second year of life would give protective efficacy almost three times greater than four doses in infancy.

**Optimal approach in extremely seasonal settings**

Due to the short transmission season, additional doses are not substantially more effective unless IPT can be given more regularly: 6 monthly doses would be similarly efficacious to 3 or 4 monthly doses (PE 81.6%, 67.1% 40.7% at 12, 15 and 24 months respectively). Six doses of bi-weekly IPT over the course of the transmission season (during infancy alone) would give protective efficacy of 95.0% in infancy, 78.8% by 15 months of age and 47.8% by two years of age.

**Table S7. Protective efficacy of IPTi and sIPT in an extremely seasonal setting**

|  | EPI-IPTi vs. seasonal IPT in infants | | EPI-IPTi vs. sIPT in children <24 months | |
| --- | --- | --- | --- | --- |
|  | Protective efficacy at 24 months | Relative PE | Protective efficacy at 24 months | Relative PE |
| IPTi (in infancy) | 15.8% | - | 15.8% | - |
| sIPT (1 dose) | 22.3% | 1.41 | 45.8% | 2.90 |
| sIPT (2 doses) | 34.7% | 2.20 | 70.9% | 4.49 |
| sIPT (3 doses) | 39.9% | 2.52 | 80.9% | 5.12 |
| sIPT (4 doses) | 40.5% | 2.55 | 81.9% | 5.18 |

The table shows protective efficacy of different number of doses of monthly seasonal IPT in comparison to four doses of IPTi in infancy; the relative protective efficacy compared to IPTi in infancy is indicated. The impact of extending the seasonal IPT schedule to also include one year old children is also shown.

**Consistency with trials from extremely seasonal settings**

These predictions are consistent with results of seasonal IPT trials. Three monthly doses of IPT with SP-AS in Niakhar gave protective efficacy of 86% in 2002 [30], and three monthly doses of SP-AQ gave an estimated protective efficacy of 93% in the same region in 2004 [31]. Protective efficacy in these studies may be higher than that predicted by the model because malaria incidence (and thus the PE) was only measured during the transmission season rather than across the whole year. Additionally, SP may be more efficacious in Senegal than Ghana, meaning that the duration of protection of SP may be underestimated.

**References**

1. Wearing HJ, Rohani P, Keeling MJ (2005) Appropriate models for the management of infectious diseases. PLoS Med 2: e174.

2. Okell LC, Drakeley CJ, Bousema T, Whitty CJ, Ghani AC (2008) Modelling the impact of artemisinin combination therapy and long-acting treatments on malaria transmission intensity. PLoS Med 5: e226; discussion e226.

3. Oduro AR, Anyorigiya T, Hodgson A, Ansah P, Anto F, et al. (2005) A randomized comparative study of chloroquine, amodiaquine and sulphadoxine-pyrimethamine for the treatment of uncomplicated malaria in Ghana. Trop Med Int Health 10: 279-284.

4. White NJ (2008) How antimalarial drug resistance affects post treatment prophylaxis. Malar J 7: 9.

5. Watkins WM, Mberu EK, Winstanley PA, Plowe CV (1997) The efficacy of antifolate antimalarial combinations in Africa: a predictive model based on pharmacodynamic and pharmacokinetic analyses. Parasitol Today 13: 459-464.

6. White NJ (2005) Intermittent presumptive treatment for malaria. PLoS Med 2: e3.

7. Cairns M, Carneiro I, Milligan P, Owusu-Agyei S, Awine T, et al. (2008) Duration of Protection against Malaria and Anaemia Provided by Intermittent Preventive Treatment in Infants in Navrongo, Ghana. PLoS ONE 3: e2227.

8. Molineaux L, Gramiccia G (1980) The Garki Project. Geneva: World Health Organisation.

9. Kitua AY, Smith T, Alonso PL, Masanja H, Urassa H, et al. (1996) Plasmodium falciparum malaria in the first year of life in an area of intense and perennial transmission. Trop Med Int Health 1: 475-484.

10. Gosling RD, Ghani AC, Deen JL, von Seidlein L, Greenwood BM, et al. (2008) Can changes in malaria transmission intensity explain prolonged protection and contribute to high protective efficacy of intermittent preventive treatment for malaria in infants? Malar J 7: 54.

11. Smith DL, Dushoff J, Snow RW, Hay SI (2005) The entomological inoculation rate and Plasmodium falciparum infection in African children. Nature 438: 492-495.

12. Sinden RE, Gilles HM (2002) The malaria parasites. In: Warrell DA, Gilles HM, editors. Essential malariology. Fourth Edition ed. London: Arnold.

13. Anto F, Tindana C, Asoala V, Awini E, Adjuik M, et al. (2004) Entomological inoculation rate in Kassena Nankana District, Ghana: Nov 2001 to October 2004 (unpublished data).

14. White NJ (2004) Antimalarial drug resistance. Journal of Clinical Investigation 113: 1084-1092.

15. Chandramohan D, Owusu-Agyei S, Carneiro I, Awine T, Amponsa-Achiano K, et al. (2005) Cluster randomised trial of intermittent preventive treatment for malaria in infants in area of high, seasonal transmission in Ghana. British Medical Journal 331: 727-733.

16. Charlwood JD, Smith T, Lyimo E, Kitua AY, Masanja H, et al. (1998) Incidence of Plasmodium falciparum infection in infants in relation to exposure to sporozoite-infected anophelines. Am J Trop Med Hyg 59: 243-251.

17. Smith T, Maire N, Dietz K, Killeen GF, Vounatsou P, et al. (2006) RELATIONSHIP BETWEEN THE ENTOMOLOGIC INOCULATION RATE AND THE FORCE OF INFECTION FOR PLASMODIUM FALCIPARUM MALARIA. Am J Trop Med Hyg 75: 11-18.

18. Beier JC, Oster CN, Onyango FK, Bales JD, Sherwood JA, et al. (1994) Plasmodium falciparum incidence relative to entomologic inoculation rates at a site proposed for testing malaria vaccines in western Kenya. Am J Trop Med Hyg 50: 529-536.

19. Dietz K, Molineaux L, Thomas A (1974) A malaria model tested in African savannah. Bulletin of the World Health Organisation 50: 347-357.

20. Epstein JE, Rao S, Williams F, Freilich D, Luke T, et al. (2007) Safety and clinical outcome of experimental challenge of human volunteers with Plasmodium falciparum-infected mosquitoes: an update. J Infect Dis 196: 145-154.

21. Smith T, Felger I, Kitua A, Tanner M, Beck HP (1999) Dynamics of multiple Plasmodium falciparum infections in infants in a highly endemic area of Tanzania. Trans R Soc Trop Med Hyg 93 Suppl 1: 35-39.

22. Smith T, Felger I, Beck HP, Tanner M (1999) Consequences of multiple infection with Plasmodium falciparum in an area of high endemicity. Parassitologia 41: 247-250.

23. Falk N, Maire N, Sama W, Owusu-Agyei S, Smith T, et al. (2006) Comparison of PCR-RFLP and Genescan-based genotyping for analyzing infection dynamics of Plasmodium falciparum. Am J Trop Med Hyg 74: 944-950.

24. Smith T, Vounatsou P (2003) Estimation of infection and recovery rates for highly polymorphic parasites when detectability is imperfect, using hidden Markov models. Stat Med 22: 1709-1724.

25. Baird JK, Owusu Agyei S, Utz GC, Koram K, Barcus MJ, et al. (2002) Seasonal malaria attack rates in infants and young children in northern Ghana. Am J Trop Med Hyg 66: 280-286.

26. Bejon P, Warimwe G, Mackintosh CL, Mackinnon MJ, Kinyanjui SM, et al. (2009) Analysis of immunity to febrile malaria in children that distinguishes immunity from lack of exposure. Infect Immun 77: 1917-1923.

27. Hviid L (2005) Naturally acquired immunity to Plasmodium falciparum malaria in Africa. Acta Tropica 95: 270-275.

28. Chandramohan D, Webster J, Smith L, Awine T, Owusu-Agyei S, et al. (2007) Is the Expanded Programme on Immunisation the most appropriate delivery system for intermittent preventive treatment of malaria in West Africa? Trop Med Int Health 12: pp 1-8.

29. Robert V, Dieng H, Lochouran L, Traore SF, Trape JF, et al. (1998) [Malaria transmission in the rural zone of Niakhar, Senegal]. Trop Med Int Health 3: 667-677.

30. Cisse B, Sokhna C, Boulanger D, Milet J, Ba EH, et al. (2006) Seasonal intermittent preventive treatment with artesunate and sulfadoxine-pyrimethamine for prevention of malaria in Senegalese children: a randomised, placebo-controlled, double-blind trial. Lancet 367: 659-667.

31. Sokhna C, Cisse B, Ba EH, Milligan P, Hallett R, et al. (2008) A Trial of the Efficacy, Safety and Impact on Drug Resistance of Four Drug Regimens for Seasonal Intermittent Preventive Treatment for Malaria in Senegalese Children. PLoS ONE 3: e1471.
